# Association between pregnancy intention and late initiation of antenatal care among pregnant women in Ethiopia: a systematic review and meta-analysis

**DOI:** 10.1186/s13643-020-01449-9

**Published:** 2020-08-20

**Authors:** Tadesse Tolossa, Ebisa Turi, Getahun Fetensa, Ginenus Fekadu, Fassikaw Kebede

**Affiliations:** 1 Department of Public Health, Institutes of Health Sciences, Wollega University, Nekemte, Ethiopia; 2 Department of Nursing, Institutes of Health Sciences, Wollega University, Nekemte, Ethiopia; 3 Department of Pharmacy, Institutes of Health Sciences, Wollega University, Nekemte, Ethiopia; 4 Department of Public Health, College of Health Sciences, Jimma University, Jimma, Ethiopia

**Keywords:** Pregnancy intention, Delayed ANC, Systematic review, Ethiopia

## Abstract

**Background:**

Antenatal care is one of the continua of reproductive health care, and inadequate antenatal care utilization results in an adverse feto-maternal outcome. Pregnancy intention is an essential factor that plays a paramount role on timing of antenatal care service. The finding of a few studies conducted on the association between pregnancy intention and late initiation of ANC among pregnant women in Ethiopia presented inconclusive. Therefore, the objective of this systematic review and meta-analysis was to determine the pooled estimate of the association between pregnancy intention and late initiation of ANC among pregnant women in Ethiopia.

**Methods:**

Both published and unpublished studies were accessed through electronic search from databases such as MEDLINE, Scopes, PubMed, CINAHL, PopLine, MedNar, Cochrane library, the JBI Library, the Web of Science, and Google Scholar. All observational studies that were conducted on the association between pregnancy intention and late initiation of ANC among pregnant women in Ethiopia were included. STATA 14.1 version was used for data analysis. A random effects model was used to estimate the pooled estimate with a 95% confidence interval (CI). The Cochrane *Q* test statistic and *I*^2^ tests were used to assess heterogeneity. Presence of publication bias was checked by funnel plots and Egger’s statistical tests.

**Results:**

A total of 670 published and unpublished studies were identified from several databases and fourteen studies fulfilled inclusion criteria and included in the meta-analysis. The overall pooled estimate indicates the odds of late initiation of antenatal care were 2.16 times higher among pregnant women who had unintended pregnancy as compared to pregnant women who had intended pregnancy (OR 2.16, 95% CI 1.62, 2.88).

**Conclusion:**

The systematic review and meta-analysis found a statistically significant effect of pregnancy intention on late initiation of antenatal care among pregnant women in Ethiopia. Increased effort should be made to improve women’s behavior towards contraceptive use through health education and counseling, especially those with unintended pregnancies. Furthermore, health education, counseling, and communication campaigns related to the timing of ANC and frequency should be promoted nationally.

## Background

Sub-Saharan African (SSA) countries contribute to the highest maternal, newborn, and child mortality, globally. Although maternal health service utilization is associated with a decrement of millions of maternal, newborn, and child death [1], globally, only two-thirds of women received four antenatal care (ANC) visits [2], 80% of live birth attended by skilled birth attendant [3], and few of them used postnatal care [4]. Maternal mortality is one of the targets of SDGs, in which the target 3.1 calls for the global maternal mortality ratio reduction below 70 deaths per 100,000 live births [5]. Strengthening maternal health service utilization is necessary to accelerate the progress of developing countries and the international community to prevent maternal and child morbidity and reach the related Sustainable Development Goals (SDG) [6].

Antenatal care is one of the continua of reproductive health care which is renowned as a vital maternal service in improving a wide range of health outcomes for women and children [7]. Timely initiation of first antenatal care visit will play an essential role for opening a doorway for maternal and child health continuum of care [1]. It can also ensure optimal health outcomes for women and children, and it is recommended that all pregnant women initiate antenatal care in the first trimester of pregnancy (early antenatal care visit of less than the first 12 weeks [7, 8]. Inadequate antenatal care utilization such as either late initiation of antenatal care or low frequency of visits results in adverse feto-maternal outcomes [9]. Utilization of at least one antenatal care visit by a skilled provider during pregnancy reduces the risk of neonatal mortality by 39% in SSA countries [10]. Accordingly, in order to decrease maternal and neonatal mortality, all pregnant women should initiate antenatal care visit during pregnancy as early as possible [6, 7, 10].

Studies showed that the majority of pregnant women begin their first ANC visit lately. A study conducted in Western Ethiopia indicated that 81.5% of women got ANC service lately [11]. Another study in Northern Ethiopia also reported that more than half of pregnant women initiated ANC after the recommended period time [12]. A systematic review and meta-analysis conducted on delayed initiation of antenatal care in Ethiopia revealed that the magnitude of delayed ANC was 64% [13].

Pregnancy intention is also another essential factor that plays a vital role on maternal health service utilization, including the late commencement of antenatal care service. Women experiencing unplanned pregnancies are more likely to delay antenatal care [14, 15]. Unplanned pregnancy is becoming a public health concern both in developed and developing countries [16, 17]. Globally, there were about 99.1 million unintended pregnancies per year in 2010–2014 in the world, and in sub-Saharan Africa, about 65 per 1000 women aged 15–44 years bear unintended pregnancy [18].

Unintended pregnancy (mistimed/unwanted in particular) is associated with adverse feto-maternal outcomes. For instance, existing evidence shows the presence of a relationship between unintended childbearing and various misbehaviors such as smoking and alcohol consumption [19]. Moreover, unintended pregnancy is associated with several adverse health outcomes such as poor women’s psychological well-being [20], maternal depression and anxiety [21], and inadequate utilization of prenatal care or skilled birth attendance [19, 22, 23]. Overall, it has been established that women who experience an unintended pregnancy are less likely than women with intended pregnancies to seek care [13–15]. Indeed, unintended/mistimed pregnancy is associated with unhealthy perinatal behaviors [19].

Even though there is a high rate of unintended pregnancies in both developed and developing countries and its potential child and maternal adverse health impact, there is a necessity to know more about the associations between pregnancy intention and late initiation of antenatal care visit. To the extent of our knowledge, there has been no systematic review and meta-analysis of pregnancy intention and its association with the late initiation of antenatal care visit. The current meta-analysis focuses on the pooled association between pregnancy intention and late initiation of antenatal care.

## Methods

### Search strategy

The Preferred Reporting Items for Systematic Reviews and Meta-Analyses (PRISMA) checklist was used to report the review [24]. Literature was downloaded to Endnote (version X7.2) to maintain and manage citations and facilitate the review process [25].

At the beginning, we checked for the presence of the existing systematic review and meta-analysis on a similar topic using the trial registries and the Cochrane library to avoid duplication. A systematic search of electronic databases such as MEDLINE, Scopes, PubMed, CINAHL, PopLine, MedNar, Cochrane library, the JBI Library, the Web of Science, and Google Scholar was done. All studies were searched using the key terms “antenatal care, late initiation, delayed initiation, early booking of ANC, pregnancy intention, unintended pregnancy, determinant factors, Ethiopia.” Addis Ababa University Digital Library and African digital library were searched to find unpublished papers [26–29]. The search was conducted from September 15, 2019, to October 30, 2019, among studies conducted until October 30, 2019. All authors participated in the search of the literature.

The pre-defined search terms were used to allow us a comprehensive search of important studies included in our review. All fields within records and Medical Subject Headings (MeSH terms) were used to help enlarge the search in advanced PubMed search. The following search strategies were adapted for the various databases using the two important Boolean operators and search engines with initial search terms “delayed initiation” OR “late initiation” OR “early initiation” AND “associated factors” OR “determinant factors” OR “predictors” AND “Antenatal care” [MeSH Terms] AND “pregnant women.”

### Selection and eligibility criteria

#### Inclusion criteria

This systematic review and meta-analysis included studies that were conducted on the association between pregnancy intention and late initiation of ANC among pregnant women in Ethiopia. We restricted our searches to human studies published in the English language. Participants were pregnant women and the review considered studies conducted with all observational study design; studies report pregnancy intention as a variable, which were written in English; and all studies are conducted in Ethiopia. All studies that were published in the form of journal articles and master’s thesis and dissertations till October 30, 2019, were included. The systematic review and meta-analysis used the PICO (Population, Intervention, Comparison, and Outcomes) framework to assess the eligibility of the articles included. The study population (P) were all pregnant women in Ethiopia, the Intervention (I) was having unintended pregnancy on their last pregnancy, the Comparison (C) was pregnant women who had intended pregnancy on their last pregnancy, and the Outcomes (O) of the interest was pregnant women who booked ANC after 12 weeks of gestational age in some studies or 16 weeks of gestation in other studies.

#### Exclusion criteria

We exclude articles that were not accessible after contacting the principal investigator two times by email. We excluded studies found only as abstract since it was difficult to access all essential information required for the analysis.

### Outcome measurement

The primary outcome variable was the association between pregnancy intention and late initiation of ANC. Pregnancy intention was measured by asking a woman’s desire about her pregnancy at the time she was pregnant (intended/unintended pregnancy). Accordingly, intention to become pregnant was categorized as intended and unintended pregnancy. Intended pregnancy is when the mother indicated that she wanted to become pregnant on their last pregnancy. Unintended pregnancy is classified into mistimed and unwanted. Both show a pregnancy that had not been wanted at the time conception occurred. Mistimed pregnancies are those pregnancies that were wanted by the woman at some time, but which occurred sooner than they were wanted, and unwanted pregnancies are all pregnancies that occurred when the woman did not want to have any more pregnancies at all [30, 31]. For this review, delay or late initiation of the first ANC visit is an initiation of the first ANC visit in public health facilities by skilled health personnel after 12 weeks of gestation in some studies and after 16 weeks of gestation in others (Table 1).

**Table 1:** Summary of included studies regarding the association between the intention of pregnancy and delayed initiation of ANC among pregnant women in Ethiopia, 2019

S.N	Author	Year	Region	Study design	Study area	Definition of late initiation of ANC	Sample size	Planned pregnancy	TOT	Unplanned pregnancy	TOT	OR (95% CI)
1	Mengesha et al .[34]	2017	SNNP	Cross-sectional	Sidama zone	ANC initiation after 12 weeks of gestation	631	377	479	99	129	0.89 (0.56, 1.41)
2	Desta et al. [35]	2018	SNNP	Cross-sectional	Hadiya zone	ANC initiation after 12 weeks of gestation	770	329	404	138	143	6.29 (2.49, 15.89)
3	Feleke, Yohannes et al. [36]	2015	SNNP	Cross-sectional	Arbaminch town	starting ANC after 16 weeks of gestation	409	208	269	130	140	3.81 (1.88, 7.70)
4	Amtatachew et al. [37]	2013	Amhara	Cross-sectional	Debre berhan town	first ANC initiation after 12 weeks of gestation	465	167	241	162	205	1.66 (1.08, 2.57)
5	Tadele Girum [38]	2016	SNNP	Cross-sectional	Dilla town	First ANC initiation after 16 weeks of gestation	362	118	271	64	91	3.07 (1.84, 5.11)
6	Belayneh et al. [39]	2014	Amhara	Cross-sectional	Gondar town	First ANC initiation after 12 weeks of gestation	369	178	332	17	37	0.74 (0.37, 1.45)
7	Wondwossen et al. [40]	2015	Tigrai	Cross-sectional	Adigrat town	First ANC initiation after 16 weeks of gestation	423	195	393	20	23	6.76 (1.98, 23.14)
8	Hussen et al. [43]	2016	SNNP	Cross-sectional	Wollaita Soddo zone	First ANC initiation after 16 weeks of gestation	256	131	197	39	58	1.03 (0.55, 1.92)
9	Kahasse et al. [45]	2017	AA	Case-control	AA	First ANC initiation after 12 weeks of gestation	402	102	348	32	54	3.50 (1.94, 6.32)
10	Yilala [29]	2015	AA	Cross-sectional	AA	First ANC initiation after 12 weeks of gestation	426	90	314	50	93	2.89 (1.79, 4.65)
11	Haileab Fekadu et al. [12]	2019	Amhara	Cross-sectional	South Gondar zone	First ANC initiation after 12 weeks of gestation	364	127	270	64	94	2.40 (1.46, 3.94)
12	Tesfalidet et al. [42]	2014	SNNP	Cross-sectional	Kembata Tembaro zone	First ANC initiation after 16 weeks of gestation	401	199	300	70	82	2.96 (1.53, 5.71)
13	Hanna G. et al. [41]	2017	AA	Cross-sectional	AA	First ANC initiation after 16 weeks of gestation	997	265	699	145	276	1.81 (1.36, 2.40)
14	Atsede et al. [44]	2018	Amhara	Cross-sectional	Debremarkos town	First ANC initiation after 16 weeks of gestation	320	67	152	40	68	1.81 (1.01, 3.23)

### Quality assessment and data abstraction

The Joanna Briggs Institute Meta-Analysis of Statistics Assessment and Review Instrument (JBI-MAStARI) was used for quality appraisal [25] (S1 Table). The quality of all the included studies was assessed by three authors (TT, GF, and FK). Any disagreement between the three authors was resolved by involving the other two authors (GF and ET) to independently assess the methodological quality to reach a consensus. Data were extracted using a standardized data extraction checklist on Microsoft Excel. Initially, duplicated literature was excluded by using Endnotes reference management. Then, studies were excluded by their titles. Next, studies that were found to be irrelevant to our study were screened by their abstracts. Full-text articles or reports were assessed for the remaining literature. Based on the preset inclusion and exclusion criteria, eligibility of the studies was evaluated. For the outcome variable (association between pregnancy intention and late initiation of ANC), data were extracted in a format of two by two tables, and then the log OR was calculated based on the findings of the original studies. The checklist for data extraction contains the title, author name, year of publication, region (the area where the study was conducted), study design, sample size, response rate, and the number of participants with the outcome (Table 1). When articles did not have adequate data, the corresponding authors of the research articles were contacted through their email.

### Heterogeneity and publication bias

Cochrane *Q* test (chi-squared statistic) and *I*^2^ test statistic on forest plot were used to check heterogeneity among the included studies. Cochrane’s *Q* statistical heterogeneity test is considered as statistically significant at *p* ≤ 0.05. *I*^2^ values of 0, 25, 50, and 75% were considered as no, low, moderate, and high degrees of heterogeneity, respectively [32]. Subgroup analysis was conducted based on the definition of ANC given by different studies. Funnel plot asymmetry was used to check publication bias. In addition, Egger’s weighted regression test was used to check publication bias [33]. A *p* value of less than 0.05 was used to declare the statistical significance of publication bias.

### Data analysis

The necessary information was extracted from each original study by using a format prepared in a Microsoft Excel spreadsheet. Then the data were exported to STATA for Windows version 14 and used to calculate the pooled effect size with 95% confidence intervals of pregnancy intention on late initiation of ANC using the DerSimonian and Laird random effects meta-analysis (random effects model). The logarithm and standard error of the odds ratio (OR) for each included study were generated using “generate” command in STATA. Meta-regression was conducted to identify the source of heterogeneity, and statistically significant results were declared in the presence of heterogeneity.

## Result

### Study selection

A systematic search of electronic databases and library catalogs identified a total of 677 published articles and three unpublished studies. Of the total published and unpublished identified studies, 97 duplicates papers were removed and 553 records were removed by reviewing titles and abstracts. The abstracts and full text of the remaining 30 studies were assessed and screened for eligibility criteria based on the outcome variable (late initiation of ANC) and 16 studies were excluded because they failed to meet the eligibility criteria (poor quality based on the selection criteria, their regression tables did not include intention of pregnancy as an independent variable).

**Fig. 1:**
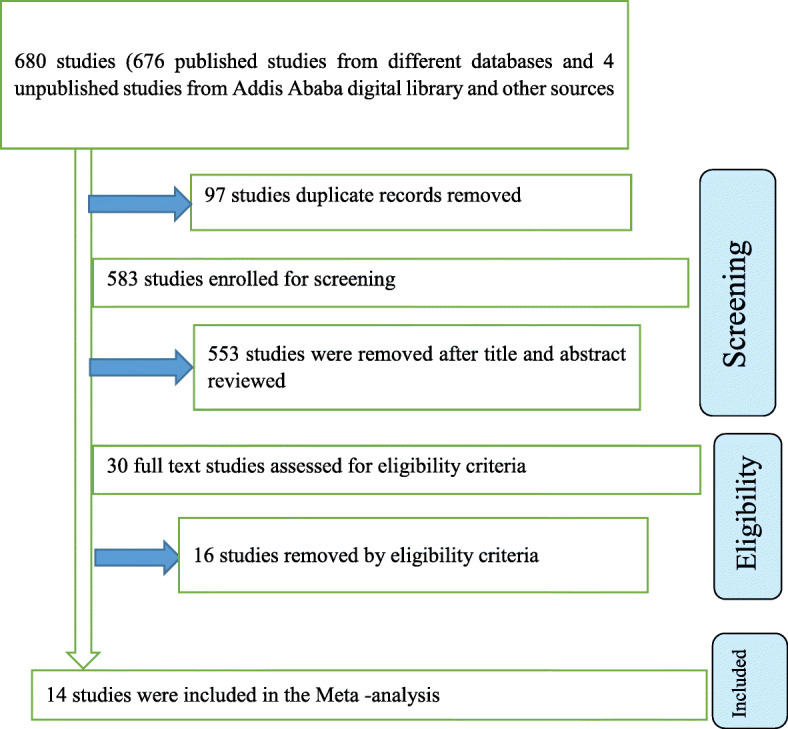
PRISMA flow diagram of included studies in the systematic review and meta-analysis of the association between intention of pregnancy and late initiation of ANC among pregnant women in Ethiopia, 2019

Finally, 14 articles which scored 7 and above on the JBI quality appraisal eligibility criteria were included in the systematic review and meta-analysis. We used the Preferred Reporting Items for Systematic Reviews and Meta-Analyses (PRISMA) flow diagram to present the systematic review overview (Fig. 1).

### Characteristics of included studies

Of the total included studies, 13 studies were cross-sectional [12, 29, 34–44] and only one study was a case-control study design [45]. They had an overall of sample size 6390 pregnant women in Ethiopia, and the study sample sizes ranged from 256 to 997 participants. The studies were conducted from 2013 to 2019 in various regions of the country. Of the fourteen studies included in the final analysis, six studies were conducted in the SNNP region [34–36, 38, 42, 43], four were conducted in the Amhara region [12, 37, 39, 44], and three were conducted in Addis Ababa [29, 41, 45]. The remaining study was conducted in Tigrai region [40].

The findings of original studies showed varied and inconclusive association between the pregnancy intention and delayed initiation of ANC. The result was a significantly positive and negative association in some studies and insignificant in other studies. Of those studies that showed a significantly positive association, the strongest association was observed in the study conducted in the Tigrai region [40] with an odds ratio of 6.77 (CI 1.98–23.15). The smallest odds ratio for the effect of pregnancy intention on late initiation of ANC (0.74) was reported in the study conducted in the Amhara region [39] (Table 1).

### Association between pregnancy intention and late initiation of ANC

In this meta-analysis, we found significant heterogeneity across studies (*I*^2^ = 73.5%, *p* = 0.00), which is an indicator to use random effects model to estimate the pooled effect of pregnancy intention on the delayed initiation of ANC reported by the fourteen studies with inverse variance.

**Fig. 2:**
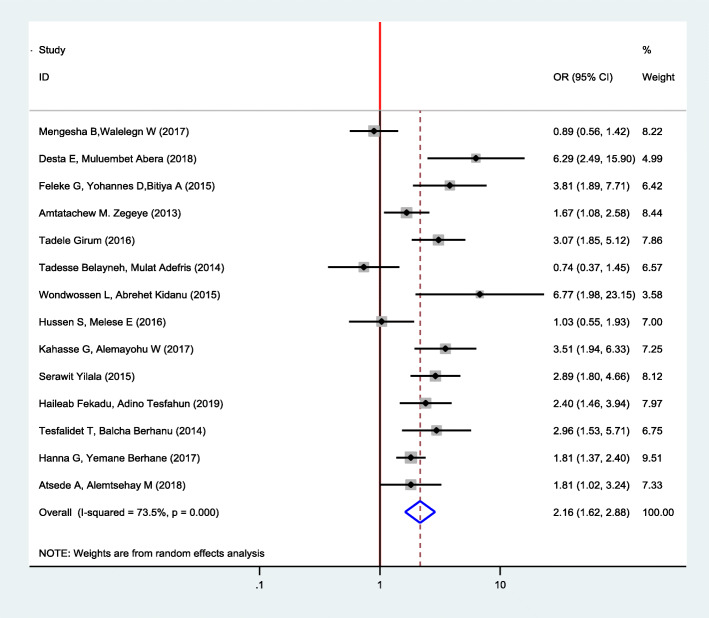
Forest plot of the pooled estimate of the association between pregnancy intention and delayed initiation of ANC among pregnant women in Ethiopia, 2019

This meta-analysis found that pregnancy intention had statistically significant effects on delayed initiation of ANC. The finding showed that there is an increased odds of delayed antenatal care among women with unintended pregnancies as compared to women with intended pregnancies (OR 2.16, 95% CI 1.62, 2.88) (Fig. 2).

**Fig. 3:**
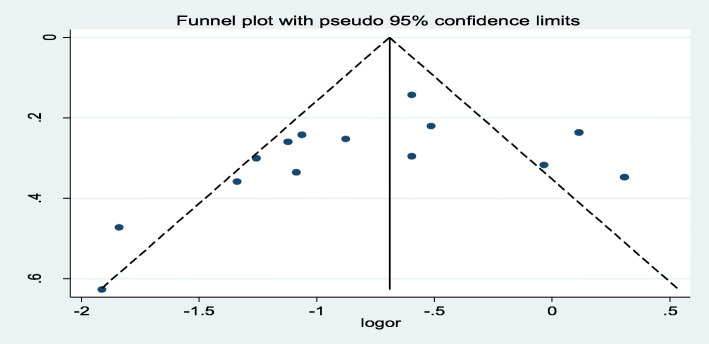
Funnel plot with 95% confidence limit of the association between pregnancy intention and delayed initiation of ANC among pregnant women in Ethiopia, 2019

To identify the possible sources of heterogeneity, different factors associated with the heterogeneity such as sample size and year of publication were computed using meta-regression models, but all of these variables were not found to be statistically significant. The presence of publication bias was assessed using a funnel plot and Egger’s test at a 5% level of significance (Fig. 3). Although visual examination of the funnel plot shows it to be asymmetric, Egger’s test was insignificant for the presence of publication bias (*p* = 0.28) (Table 2).

**Table 2:** Related factors with heterogeneity of association between pregnancy intention and delayed initiation of ANC among pregnant women in Ethiopia, 2019

Variables	Coefficients	***p*** value
Publication year	0.0404114	0.719
Sample size	0.0000683	0.943

**Fig. 4:**
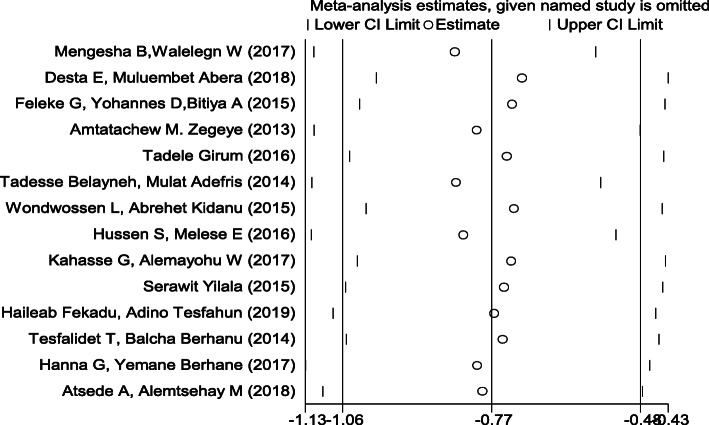
Sensitivity analysis for single study influence of the association between pregnancy intention and delayed initiation of ANC among pregnant women in Ethiopia, 2019

Sensitivity analysis of the studies was done to test the effect of a single study on the pooled result of the remaining studies using random effect model. We found no strong evidence for influence of individual study on remaining studies (Fig. 4).

**Fig. 5:**
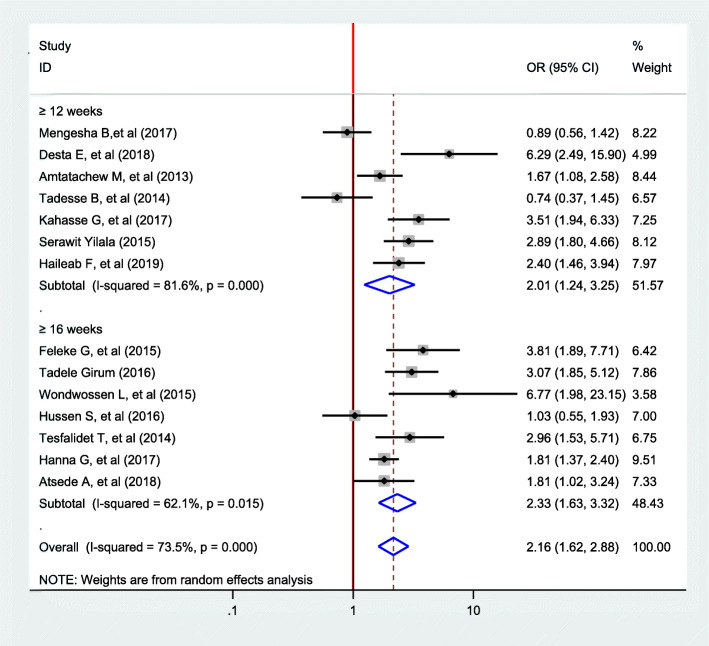
Subgroup analysis of the association between pregnancy intention and delayed initiation of ANC in Ethiopia, 2019

Subgroup analysis was conducted based on the definition of late initiation of ANC given by different studies. Accordingly, the odds of delayed ANC initiation among women with unintended pregnancy was 2.10 times higher as compared to women with intended pregnancy with studies used 12 weeks definition of delayed initiation of ANC (OR 2.10, 95% CI 1.23, 3.24). The odds of delayed ANC initiation women with unintended pregnancy was 2.33 times higher as compared to women with intended pregnancy with studies used 16 weeks definition of delayed initiation of ANC (OR 2.33, 95% CI 1.63, 3.32) (Fig. 5).

## Discussion

Timely initiation and continuously attending ANC service can improve maternal and child health outcomes. This is very important in a low-income country such as Ethiopia where the health condition of the mother is very poor. The current systematic review and meta-analysis estimated the pooled effect of pregnancy intention on delayed initiation of ANC among pregnant women in Ethiopia.

The result of this study found that pregnancy intention had statistically a significant effect on late initiation of ANC among pregnant women. The overall pooled estimate of this analysis indicated the odds of delayed initiation of ANC among women who experienced unintended pregnancy were higher than their counterparts. Delaying time of ANC initiation endangers both maternal and fetal health. This is due to the fact that a delay in ANC commencement decreases the full benefit of women from effective ANC follow-up and timely initiation of ANC [46]. It is believed that intended pregnancies are more cared for by pregnant women and their spouses; this enables women to book for ANC timely.

The finding is consistent with a previous systematic review and meta-analysis conducted in Ethiopia on overall factors associated with late initiation of ANC, in which women with intended pregnancy were less likely to delay their ANC initiation [13]. The possible reason for delayed initiation of ANC among women with unintended pregnancy may be the absence of good health care behavior due to lack of family or social support. Furthermore, the finding of this meta-analysis is similar to studies conducted in Tanzania in which unintended pregnancy decreased early initiation of ANC due to late recognition of pregnancy and socioeconomic factors [22, 46].

A study conducted in Kenya showed that women who reported unintended pregnancy were either less likely to receive antenatal care or more likely to delay ANC initiation [47], which is consistent with our finding. Another study from South Africa also reported that unintended pregnancy, which was attributed mainly to failure of family planning, was a significant predictor of late initiation of ANC [48–50]. This might be if pregnancy is unintended pregnancy, the intention to care for pregnancy is low and the effort to hide a pregnancy is very high because of social fear and custom of the community. Similarly, the finding of the current study is consistent with a national-level study from Rwanda, which stated women with unintended pregnancy are less likely to attend pregnancy and more likely in late initiation of ANC [51]. The result from Zambia and Myanmar reveals the same finding in which women who fell pregnant unintentionally had a higher odds of starting ANC late, which is consistent with our current result [52, 53].

Women with unintended pregnancy may delay ANC initiation due to lack of knowledge, lack of decision making power, lack of money, or cultural factors [46]. Unintended pregnancies are also related to social and cultural determinants of health-seeking behaviors such as ineffective use of family planning, sexual violence, and barriers to access to health care; these all may be associated with late initiation of ANC [22].

The strength of this study is that various databases were used to search literature, and both published and unpublished studies were included in the study. This study has some limitations. First, most of the included studies in the final analysis were cross-sectional study design which may decrease causal conclusion between pregnancy intention and delayed initiation of ANC.

## Conclusion

This systematic review and meta-analysis demonstrated that women’s pregnancy intention was associated with late commencement of antenatal care in Ethiopia. To reduce unintended pregnancy, the reproductive health services that focus on strengthening the women utilization of family planning are demanded. Increased effort should be made to improve women’s behavior towards contraceptive use through health education and counseling, especially those with unintended pregnancies. Furthermore, health education, counseling, and communication campaigns related to the timing of ANC and frequency should be promoted nationally.

## Supplementary information

**Additional file 1: Table S1.** JBI Quality Assessment tool.

**Additional file 2: Table S2.** Result of database search.

## Data Availability

All data analyzed during this study are included in the manuscript.

## Abbreviations

ANC: Antenatal care
CI: Confidence interval
SSA: Sub-Saharan Africa
SDG: Sustainable Development Goals
OR: Odds ratio
SNNP: Southern Nation Nationalities and People
WHO: World Health Organization
