# Supplementary material for: Association between pregnancy intention and late initiation of antenatal care among pregnant women in Ethiopia: a systematic review and meta-analysis
Source: Syst Rev. 2020 Aug 20;9:191. doi: 10.1186/s13643-020-01449-9 (PMC7441676; doi:10.1186/s13643-020-01449-9)
Supplement: Supplementary file 1 — Additional file 1: Table S1. JBI Quality Assessment tool. [file 13643_2020_1449_MOESM1_ESM.doc]

**S3 Table: JBI MAStARI Critical Appraisal Checklist for Analytical Cross Sectional Studies**

Reviewer Date

Author Year Record Number---------

|  | Yes | No | Unclear | Not applicable |
| --- | --- | --- | --- | --- |
| 1. Were the criteria for inclusion in the sample clearly defined? |  |  |  |  |
| 1. Were the study subjects and the setting described in detail? |  |  |  |  |
| 1. Was the exposure measured in a valid and reliable way? |  |  |  |  |
| 1. Were objective, standard criteria used for measurement of the condition? |  |  |  |  |
| 1. Were confounding factors identified? |  |  |  |  |
| 1. Were strategies to deal with confounding factors stated? |  |  |  |  |
| 1. Were the outcomes measured in a valid and reliable way? |  |  |  |  |
| 1. Was appropriate statistical analysis used? |  |  |  |  |

Overall appraisal: -------- Include------- Exclude------- Seek further info--------

Comments (Including reason for exclusion)
