# Supplementary material for: Association between pregnancy intention and late initiation of antenatal care among pregnant women in Ethiopia: a systematic review and meta-analysis
Source: Syst Rev. 2020 Aug 20;9:191. doi: 10.1186/s13643-020-01449-9 (PMC7441676; doi:10.1186/s13643-020-01449-9)
Supplement: Supplementary file 2 — Additional file 2: Table S2. Result of database search. [file 13643_2020_1449_MOESM2_ESM.docx]

| **Databases** | **Searching terms** | **Number of studies** |
| --- | --- | --- |
| **PubMed** | ((((((((((delayed initiation) OR late initiation) OR early initiation) AND associated factors) OR determinant factors) OR predictors) AND Antenatal care[MeSH Terms]) AND pregnant women) | **363** |
| **From other databases** |  | **313** |
| **Digital library** |  | **4** |
| **Total retrieved articles** |  | **680** |
| **Final full text relevant to our review** |  | **14** |
